# Plant peptide hormones: three decades of discovery and emerging paradigms

**DOI:** 10.1093/plphys/kiag699

**Published:** 2026-09-23

**Authors:** Keiko U Torii

**Affiliations:** Department of Molecular Biosciences and Howard Hughes Medical Institute, The University of Texas at Austin, 2506 Speedway, Austin, TX 78712, United States

## Abstract

Peptide hormones have reshaped our understanding of intercellular signaling in plants. Once considered absent in plants, peptide-based signaling pathways emerged as key regulators of plant growth, development, environmental responses, and immunity. A paradigm shift occurred with the discoveries of systemin and phytosulfokine (PSK) in the 1990s, as well as the identification of receptor-like kinases regulating important biological processes. Over the past 3 decades, a repertoire of peptide hormone families has been expanded enormously, with deeper understandings of peptide modifications, cleavage, modes of action, receptor binding, and signal transduction. In this review, I highlight selected peptide hormone families–– systemin, PSK, INFLORESCENCE DEFICIENT IN ABSCISSION (IDA)/IDA-LIKES, CLAVATA3/EMBRYO SURROUNDING REGION, and EPIDERMAL PATTERNING FACTORs (EPF)/EPF-LIKES–– and discuss how individual discoveries of the peptides and their cognate receptors have shaped emerging concepts in plant peptide signaling. To celebrate this Centennial Issue, emphasis will be given to contributions published in *Plant Physiology*. Finally, I discuss how expanding genomic resources and advanced technologies will accelerate the discovery and engineering of peptide signaling pathways to enhance plant resilience.

## Introduction

The past century has witnessed unprecedented breakthroughs in science, including plant physiology. Advances in biochemistry, genetics, and molecular biology during the mid- to late 20th century have challenged and revised long-held paradigms in biology. One such dogma was that plants, unlike animals, do not have peptide hormones (indeed, as a college student in the 1980s, I learned in Plant Physiology class that plants only produce small chemical hormones—auxin, cytokinin, gibberellins, abscisic acid, and ethylene). In the 1990s, searches for biologically active factors, one inducing defense responses in Solanaceae and other promoting proliferation of Asparagus culture cells, led to the identification of small peptides––18-amino-acid systemin peptides from Solanaceae and a 5-amino-acid sulfated peptide, phytosulfokine (PSK), respectively (Pearce et al. 1991; Matsubayashi and Sakagami 1996). During the same period, molecular-genetic studies, starting from mutant phenotypes, identified receptor-like kinases (RLKs) as regulators of defense response and development in Arabidopsis, maize, and rice (Song et al. 1995; Becraft et al. 1996; Torii et al. 1996; Clark et al. 1997). These discoveries led researchers to predict that peptide growth regulators may be perceived by RLKs, therefore envisioning that plants, like animals, make use of peptide-receptor signaling pathways to regulate development and environmental response (Marx 1996; Wilson et al. 1997).

Now, 30 years later, it is firmly established that peptide hormones and their receptors are crucial and versatile regulators of growth, development, stress and defense response, and more. In this centennial celebration of *Plant Physiology*, I review major discoveries about selected peptide hormones and their receptors and highlight how *Plant Physiology* has provided a key platform for publishing these landmark studies. For this purpose, rather than providing a comprehensive list of known plant peptide hormones, I focus on 5 peptide families (systemin, PSK, INFLORESCENCE DEFICIENT IN ABSCISSION [IDA]/INFLORESCENCE DEFICIENT IN ABSCISSION-LIKE peptides [IDLs], CLAVATA3/EMBRYO SURROUNDING REGION-RELATED peptides [CLEs], and EPIDERMAL PATTERNING FACTOR [EPF]/EPIDERMAL PATTERNING FACTOR-LIKE peptides [EPFLs]) to describe how they were discovered and what conceptual breakthroughs were brought through their studies in plant biology (see Fig. 1 for a historical timeline). Careful choices are made on the peptide hormone families to be discussed, as it is impossible to cover the entire topic. Specifically, I follow the format of the *Plant Physiology* perspective article, published in 2001 by Clarence Ryan and Gregory Pearce, who described 6 peptide hormones known back then: systemin, PSK, RAPID ALKALINIZATION FACTOR (RALF), S-locus protein 11, CLAVATA3 (CLV3), and early nodulin 40 (Ryan and Pearce 2001). One can easily grasp how the world of plant peptide hormones has exploded since then. Lastly, I discuss how increasing genome information and emerging technologies will advance the future directions of plant peptide hormone research with the goal of understanding and manipulating plant resilience.

**Figure 1:**
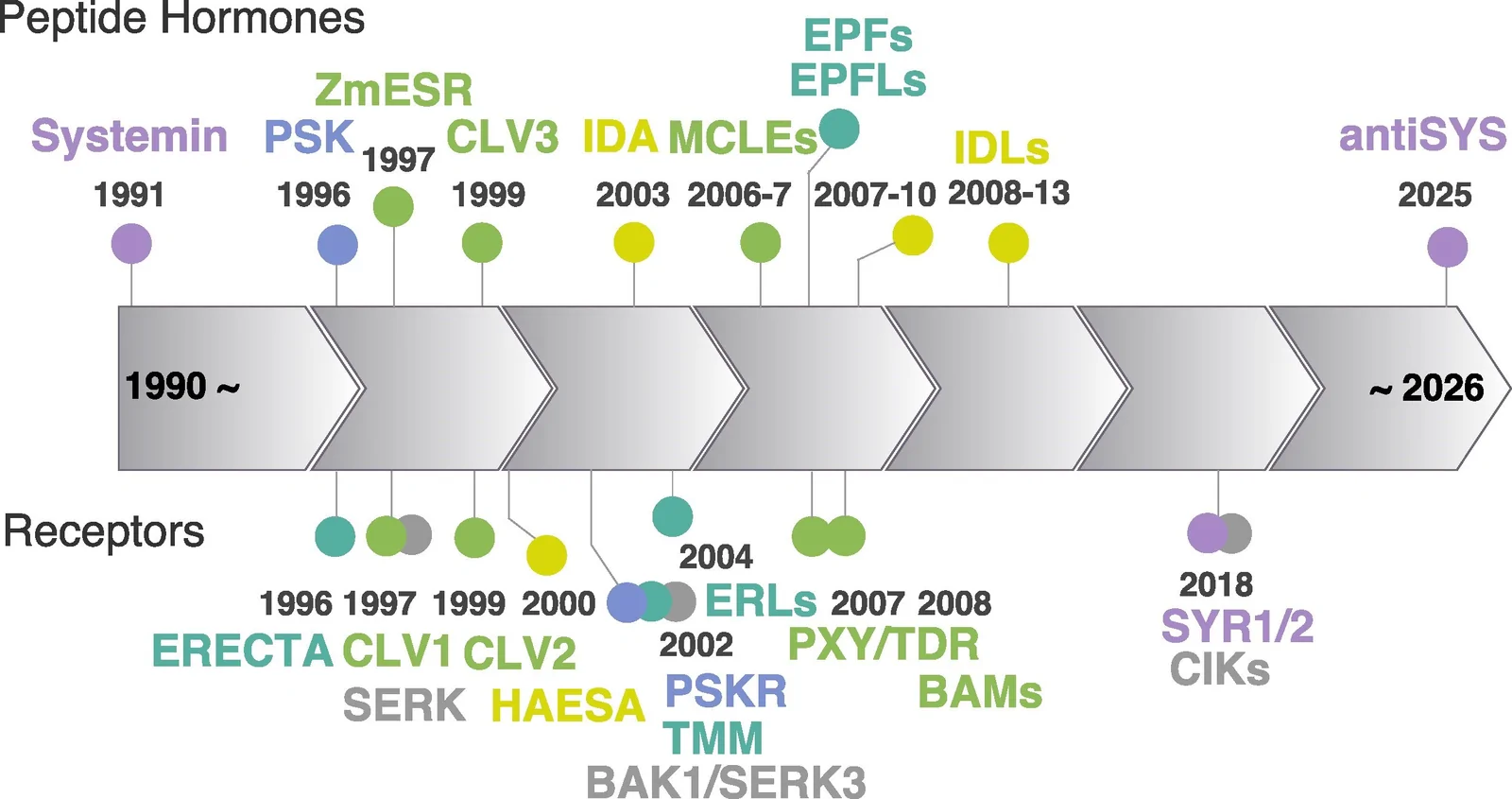
Historical timeline of plant peptide hormone research in the past 3 decades. Shown is the timeline from 1990 to 2026 (gray bars), indicating the years in which the 5 featured peptide hormone families and their corresponding receptors were discovered. Dots: systemin (lilac), PSK (indigo), IDA/IDLs (lime yellow), CLEs (yellow green), and EPFs/EPFLs (teal). Their corresponding primary receptors are color-coded to match the peptide hormones. Coreceptors are in gray. The color codes match with those in Figure 3.

## The basics of plant peptide hormones

The majority of peptide hormones are secreted into the apoplast to mediate cell–cell signaling. However, some peptide hormones are proteolytically released into the apoplastic space upon tissue or cellular damage, where they function as damage-associated molecular patterns to activate a defense response. Representative examples include systemin and plant elicitor peptide 1 (PEP1) (Tanaka and Heil 2021). Based on the structural features of mature peptides, plant peptide hormones can be classified into distinct classes: small peptides (∼20 amino acids) that are proteolytically cleaved and are often, but not always, posttranslationally modified (eg tyrosine sulfation and proline hydroxylation) and cysteine-rich peptides (CRPs), which adopt complex structures via intramolecular disulfide bond formation (Marshall et al. 2011; Matsubayashi 2014) (Fig. 2). The biological functions of plant peptide hormones are diverse, including cell growth, cell proliferation, cell fate specification, developmental processes throughout the plant life cycle, pollen-pistil recognition, pollen-tube guidance, cellular barriers, integrity sensing, nutrient sensing, symbiosis, plant parasitism, and defense and immune response (Richardson and Torii 2013; Matsubayashi 2014; Yamaguchi et al. 2016; Hou et al. 2021; Kim et al. 2021; Debnath et al. 2026; van Wullen et al. 2026; Zecua-Ramirez et al. 2026).

**Figure 2:**
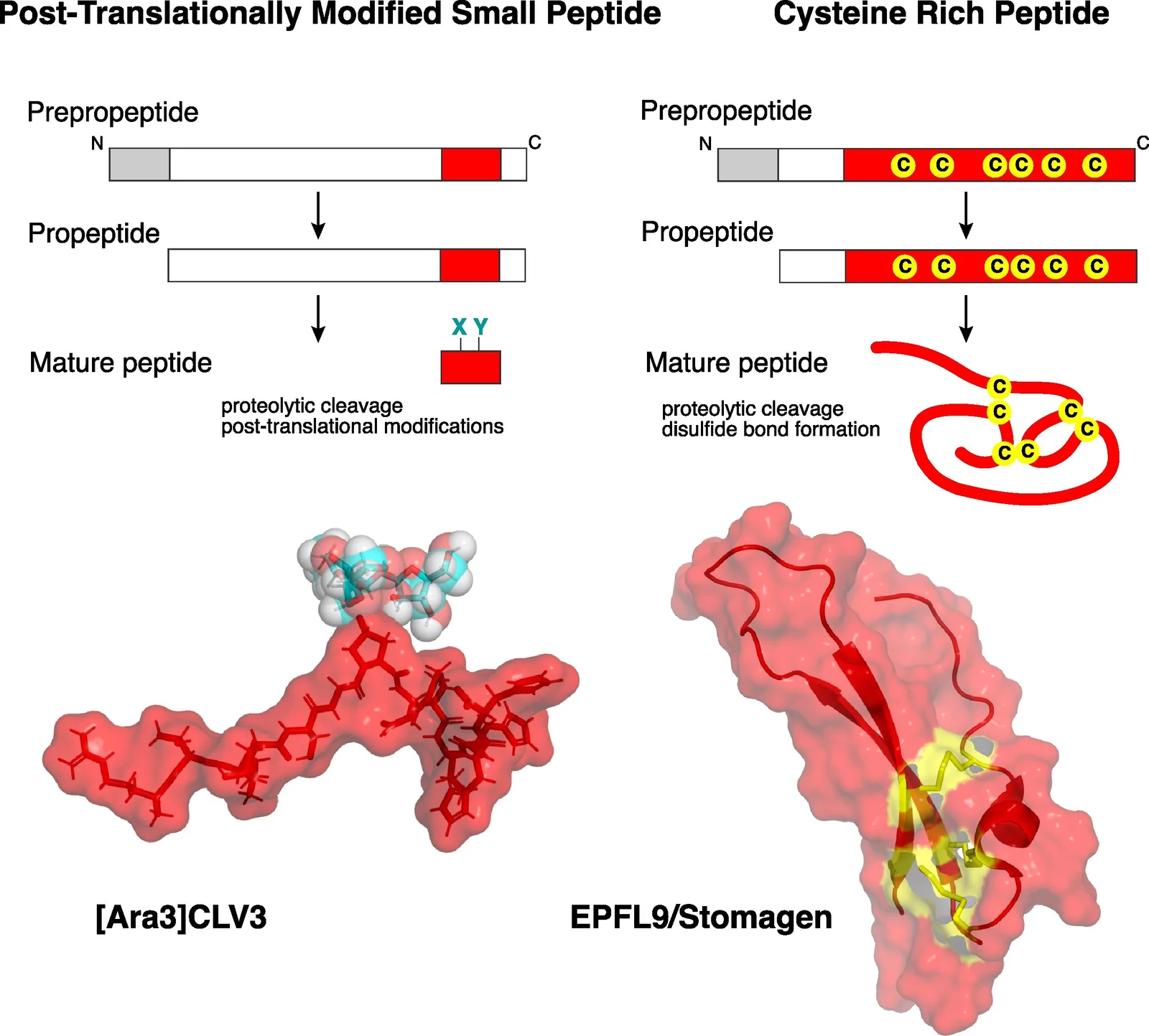
Classification of plant peptide hormones and their structural features. Plant peptide hormones can be classified into distinct classes: small posttranslationally modified peptides (left) and CRPs (right). (Top left) For posttranslationally modified peptides, their prepropeptide will be cleaved into propeptide and posttranslational modifications (eg tyrosine sulfation, proline hydroxylation, and hydroxyproline arabinosylation) (X, Y in teal). Note that not all small peptides are posttranslationally modified, and some peptides lack a signal sequence but instead become cleaved upon cell damage. (Top right) A CRP is formed through proteolytic cleavage of prepropeptide and subsequent disulfide bond formation through conserved cysteine residues (yellow). (Bottom left) The structure of a mature [Ara3]CLV3 peptide as an example of a posttranslationally modified small peptide. A solution NMR structure of the 13-CLV3 peptide (red) modified by hydroxyproline triarabisynolation (cyan) in stick representation, superimposed with a surface representation. The average (minimized) structure was generated from the top 5 solution structures. The NMR data are courtesy of Yoshikatsu Matsubayashi (published in Shinohara and Matsubayashi 2013; credit: Krishna Mohan Sepuru). (Bottom right) Structure of EPFL9/Stomagen as an example of CRPs. A solution NMR structure of mature EPFL9/Stomagen peptide (red) (PDB ID: 2LIY) in a ribbon diagram superimposed with surface representation. The cysteine residues forming disulfide bonds are highlighted in yellow (credit: Krishna Mohan Sepuru).

These peptide hormones, whether secreted or released during cellular damage, mediate intercellular communication. The important question is how far plant peptide hormones move and influence the response in target cells. Interestingly, small-secreted peptides can function as local (autocrine), short-distance (juxtacrine or paracrine), or long-distance, root-to-shoot signals. A great example is the CLE peptide family (see next section). Its founding member, CLV3, restricts stem cell homeostasis by repressing a transcription factor, WUSCHEL, in the adjacent cell populations (Fletcher et al. 1999; Brand et al. 2000). CLE5, on the other hand, is expressed in stomatal guard cells and locally elicits rapid stomatal closure upon drought (Shimotohno et al. 2025). In contrast, CLE25 mediates long-distance signals from root to shoot upon water deprivation to trigger a stomatal closure response (Takahashi et al. 2018). Larger CRPs thus far have been shown to function as a short-range signal, either in a juxtacrine or autocrine manner (Zeng et al. 2020; Bai and Torii 2026).

Thus far, cognate receptors for plant peptide hormones belong to the RLK and receptor-like protein (RLP) superfamily; a large phylogenetic study was published in *Plant Physiology* (Shiu and Bleecker 2003). An overwhelming majority of peptide hormone receptors belong to extracellular leucine-rich repeat (LRR) RLKs. Indeed, primary receptors for all peptide hormones discussed in detail in the next section (systemin, PSK, IDA/IDLs, CLEs, and EPF/EPFLs) are LRR-RLKs (Fig. 3). Many such LRR-RLKs form ligand-induced receptor heterodimers with a universal coreceptor family, SOMATIC EMBRYOGENESIS RECEPTOR KINASEs (SERKs) including BRASSINOSTEROID INSENSITIVE1 (BRI1)-ASSOCIATED KINASE1 (BAK1)/SERK3 (Schmidt et al. 1997; Li et al. 2002; Nam and Li, 2002) (Fig. 1). It is important to mention that some of the key peptide hormones are perceived by the different classes of RLKs. For example, RALF peptide receptors belong to *Catharanthus roseus* receptor-like kinase 1-like malectin-like RLKs, including FERONIA (Lu et al. 2025). Self-incompatibility determinant CRPs, such as S-locus protein 11, bind to the S-receptor kinases with an extracellular S domain (Takayama et al. 2001). Additionally, some plant peptides may function without being specifically perceived by the receptors. While this review is not focused on the mechanisms of receptor activation and downstream signal transduction pathways, their conserved and unique roles and crosstalk further emphasize the importance of peptide hormone signaling in plants.

**Figure 3:**
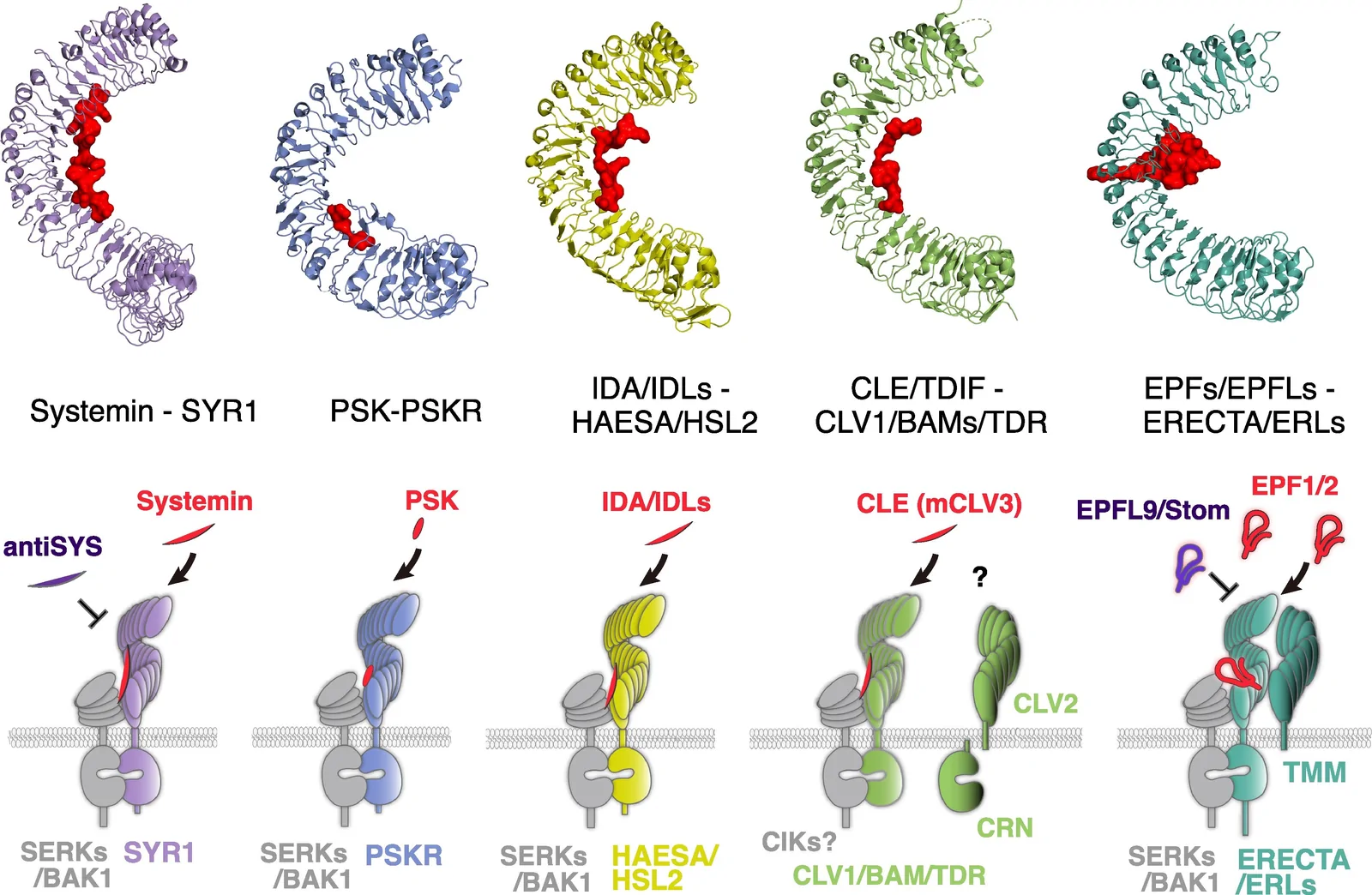
Structural basis of peptide hormone perception and receptor complex formation. (Top) Structures of the peptides (surface representations in red) bound to the LRR domain of the corresponding receptors shown as ribbon diagrams. From left, systemin bound to tomato SYR1 (lilac), PSK bound to PSK receptor (indigo) (PDB ID: 4Z63), IDA bound to HAESA (lime yellow; PDB ID: 5IXT), TDIF bound to TDIF receptor (TDR) (yellow green; PDB ID: 5GR9), and EPFL4 bound to ERL2 (teal; PDB ID: 5XKN). Structures of the peptide-receptor pairs, except for systemin and SYR1, have been solved and downloaded from the PDB site for visualization. The systemin-SYR1 structural model was generated using AlphaFold3. The tomato systemin (UniProt: P27058) was used to model the ligand-receptor complex (credit: Krishna Mohan Sepuru). (Bottom) Diagram showing the receptor complex upon perception of peptide ligands (red). The diagram includes coreceptors SERKs/BAK and CIKs (gray) as well as auxiliary receptors. From left: systemin, SYR1 (lilac) receptor complex; PSK, phytosulfokine receptor complex (indigo); IDA/IDLs, HAESA/HSL2 (lime yellow) receptor complex; CLE/tracheary element differentiation inhibitory factor, CLV1/BAMs/TDR (yellow green) receptor complex with CLV2 and CORYNE; EPFs/EPFLs, ERECTA/ERLs (teal) with TMM. ERECTA/ERLs outside the context of stomatal development form an active receptor complex without TMM.

## Plant peptide hormones: case studies

### Systemin

Systemin is the first peptide hormone identified from plants in 1991 through several decades of work by the late Clarence (Bud) Ryan, who sought mobile factors that elicit the induction of protease inhibitors against herbivores/pathogens upon Solanaceae leaf wounding (Ryan 1968, 1968, 1974). Through subsequent biochemical purification (from 27 kg of young tomato leaves from wounded plants), the causal factor systemin was identified as an 18-amino-acid soluble polypeptide [AVQSKPPSKRDPPKMQTD] (Pearce et al. 1991) (Fig. 1). Chemically synthesized systemin peptide fully elicited a wound response at the femtomolar level. Structure-function analyses revealed that all 18 amino acids are required for full activity (Pearce et al. 1993). Subsequently, a full-length gene encoding 200-amino-acid prosystemin was identified, indicating that mature systemin is produced via proteolytic cleavage (McGurl et al. 1992; McGurl and Ryan 1992).

The discovery of systemin established a new paradigm that plants produce mobile, endogenous peptide hormones to elicit defense responses. The repertoire of such peptides has expanded to include PEPs, RALFs, and pathogen-associated molecular pattern–induced secreted peptides (Hou et al. 2019). Many act as damage-associated molecular patterns to amplify immune signaling, whereas others additionally modulate plant growth, development, metabolism, and behaviors. Those multifaceted peptide hormones are collectively termed “phytocytokines,” with conceptual analogies to cytokines in humans (Hou et al. 2021).

Identifying a receptor for systemin was the next major step. In the 1990s and 2000s, several regulators of cell–cell signaling and defense/immunity response turned out to be LRR-RLKs (eg ERECTA, CLAVATA1 [CLV1], XA21 disease-resistance receptor, FLAGELLIN SENSING 2) (Torii 2000). As such, it came as no surprise that a biochemical approach using radiolabeled systemin identified an LRR-RLK of 160 kDa (systemin receptor 160 [SR160]) from a membrane fraction of tomato suspension culture cells (Scheer and Ryan 2002). The real surprise was that SR160 highly resembles the brassinosteroid receptor, BRASSINOSTEROID INSENSITIVE1 (BRI1) (Li and Chory 1997), thus fueling interest in a possibility that Solanaceae may have evolved a dual receptor for both steroid and peptide hormones. Later genetic studies, however, clarified that the tomato *bri1* mutant is fully responsive to systemin (Holton et al. 2007; Lanfermeijer et al. 2008). Then what is the systemin receptor? Using tomato introgression lines generated from cultivated tomato and a systemin-insensitive wild relative, George Felix and colleagues identified the LRR-RLKs SYSTEMIN RECEPTOR1 (SYR1) and SYSTEMIN RECEPTOR2 (SYR2) as systemin receptors, with SYR1 exhibiting high-affinity binding to systemin (Wang et al. 2018) (Figs. 1 and 3). Importantly, the introduction of SYR1 (and SYR2) was able to confer systemin responsiveness in tobacco and Arabidopsis, which do not otherwise sense the systemin peptide. In contrast, SR160 failed to confer systemin response to these heterologous plant systems (Wang et al. 2018). The quest for the systemin receptor is a compelling example of scientific discovery, in which hypotheses are rigorously tested, refined, and revised.

Unlike pathogen-derived peptides (pathogen-associated molecular patterns), phytocytokines like systemin are produced by plants themselves. How could plants prevent inappropriate activation of the defense response? A recent study revealed that tomato leaves produce a systemin antagonist, the 16-amino-acid polypeptide antiSYS (Wang et al. 2025). AntiSYS prevents the systemin response by effectively competing with systemin for receptor binding to SYR1 at an IC_50_ in the nanomolar range (Wang et al. 2025). Interestingly, tomato genome sequence analysis further revealed that the *pro-antiSYS* gene is situated within the gene cluster of 4 systemin paralogues (*pro-SYS2* ∼ *pro-SYS5*). How does antiSYS prevent systemin signaling? Further biochemical analysis revealed that antiSYS prevents the activation of SYR1 by inhibiting the recruitment of a universal coreceptor, SERKs/BAK (Wang et al. 2025) (Fig. 3). Strikingly, removal of the *pro-antiSYS* gene from tomato by CRISPR-Cas9 genome editing conferred severe growth retardation, developmental anomalies, and an autoimmune syndrome, suggesting the uncontrollable activation of phytocytokine defense signaling (Wang et al. 2025). Fascinatingly, the low-affinity binding systemin receptor, SYR2, was further shown to act as an antagonistic receptor to SYR1: whereas SYR1 tightly binds systemin to initiate defense signaling, SYR2 attenuates signal transduction by competing with SYR1 for co-receptor SERK3 heterodimerization (Zhou et al. 2025). Thus, the systemin-SYR peptide hormone-receptor system involves signal antagonism both at the ligand and receptor levels. Taken together, 3 decades of study about systemin have uncovered plant-produced peptide hormones as a crucial coordinator of plant growth-defense tradeoffs and continue to reveal remarkable modes of action of plant peptide hormone signaling.

### Phytosulfokine

Plant physiologists in the early days observed a phenomenon known as “conditioning.” Plant suspension culture cells do not proliferate if their density is too low. However, adding “conditioned media”—filtered media from densely growing cell culture—can confer active cell proliferation of low-density culture cells, indicating the presence of some growth-promoting factors (Stuart and Street 1969). Yoshikatsu Matsubayashi and colleagues successfully purified such a factor from conditioned media derived from asparagus and rice suspension cell cultures, and determined its identity as tyrosine-sulfated pentapeptide [Y(SO_3_H)-I-Y(SO_3_H)-T-Q], named phytosulfokine α (PSK-α) (Matsubayashi and Sakagami 1996; Matsubayashi et al. 1997) (Fig. 1). Chemically synthesized PSK-α exhibited potent mitogenic activity (Matsubayashi and Sakagami 1996), thus establishing PSK as the first plant peptide growth hormone. The modification of tyrosine likely renders the mature peptide stable and active (Kaufmann and Sauter 2019). The corresponding receptor for PSK was identified biochemically by radiolabeled PSK ligand-based affinity chromatography using membrane fractions from carrot culture cells (Matsubayashi et al. 2002). Remarkably, the PSK receptor turned out to be yet another LRR-RLK (Matsubayashi et al. 2002) (Figs. 1 and 3), further emphasizing the versatile roles for LRR-RLKs in peptide hormone signaling in plants. Later structural analysis revealed that PSK binding to the PSK receptor stabilizes the receptor structure, allowing recruitment of a coreceptor SERK (Wang et al. 2015) (Fig. 3).

Leveraging the full-genome sequence of Arabidopsis released in 2000, these researchers identified 4 genes encoding *AtPSK1-4*, the full-length PSK prepropeptides, a landmark finding published in *Plant Physiology* (Yang et al. 2001). Interestingly, Arabidopsis and rice full-length PSKs share limited sequence similarity outside of the mature peptide, implying that mature PSK activities from different precursors might be tightly regulated at the level of proteolytic cleavages. Despite PSK's potent activities in promoting cell dedifferentiation and proliferation (Matsubayashi and Sakagami 1996; Matsubayashi et al. 1999), the in vivo developmental functions of PSKs remained somewhat ambiguous. It has been shown that PSK-α promotes root growth and coordinates cell proliferation during wounding or stress response, including reports in *Plant Physiology* (Kutschmar et al. 2009; Motose et al. 2009; Loivamaki et al. 2010).

The discovery of PSK revealed that plants produce a peptide growth hormone, thereby expanding the repertoire of peptide hormones beyond stress/damage response. Furthermore, a series of studies by Matsubayashi et al. underscores the power of classic biochemical purification as an alternative approach to genetics, especially when genome information is scarce or genetic redundancies hamper phenotypic screens. The identity of PSK-α as a tyrosine-sulfated peptide hinted that other peptide hormones may also be modified by tyrosine sulfation. An enzyme responsible for PSK-α tyrosine sulfation, tyrosylprotein sulfotransferase, was biochemically purified from Arabidopsis (Komori et al. 2009). Strikingly, the loss-of-function *tpst* mutants exhibit severe pleiotropic defects in growth and development, far beyond the phenotype of *psk* and *pskr* mutants (Komori et al. 2009). This observation indicates the presence of additional peptide hormones modified by tyrosylprotein sulfotransferase. In fact, tyrosine-sulfated peptide families are widespread, including ROOT MERISTEM GROWTH FACTORs (RGFs; also known as GOLVENs and CLE-LIKEs), which are required for a homeostasis of the root stem cell niche, PLANT PEPTIDE CONTAINING SULFATED TYROSINEs (PSYs), which coordinate plant growth-stress response, and CASPARIAN STRIP INTEGRITY FACTORs (CIFs), which mediate diffusion barrier formation (Doblas et al. 2017; Nakayama et al. 2017; Kaufmann and Sauter 2019; Ogawa-Ohnishi et al. 2022). While not all plant peptide hormones are posttranslationally modified, the work expanded this concept to diverse modifications, such as hydroxyprolination and arabinosylation found in CLE-family peptides (Ohyama et al. 2009) (Fig. 2).

#### Inflorescence deficient in abscission

IDA and IDLs are a family of peptide hormones genetically identified by Melinka Butenko and colleagues as a gene required for floral organ abscission (Butenko et al. 2003) (Fig. 1). Floral petals of loss-of-function *ida* mutants stay attached to siliques due to defective formation of the abscission zone, and conversely, elevated *IDA* expression exaggerates the abscission zone. The full-length IDA is a small, secreted protein of 77 amino acids. IDA and the 5 related paralogs, IDL1-IDL5, share a conserved C-terminal 20-amino-acid motif, named the extended PIP motif with a conserved Pro-Ile-Pro sequence. A synthetic IDA extended PIP peptide triggers floral abscission (Stenvik et al. 2008), indicating that, like other small peptide hormones (eg PSK, CLE, RGF, PSY), cleaved C-terminal fragments function as mature, bioactive IDA/IDL peptides. IDL1 is required for cell separation during the emergence of lateral root primordia (Kumpf et al. 2013); thus, IDA/IDL peptide signaling promotes cell separation in the broader context of development. The corresponding receptor for IDA, HAESA (HAE), and its paralog HAESA-LIKE2 (HSL2) LRR-RLKs, were also identified genetically by John Walker and colleagues as a mutant phenotype defective in floral abscission (for HAE) (Jinn et al. 2000) (Fig. 1). HAE/HSL2 together regulate cell separation during lateral root primordia (Kumpf et al. 2013). Like many other ligand-binding LRR-RLKs, HAE/HSL form an active receptor heterodimer complex with SERKs/BAK upon ligand perception (Santiago et al. 2016) (Fig. 3).

Studies of IDA/IDL and HAE/HSL2 peptide-receptor pair interactions further broadened the concept of peptide hormones mediating both development and defense/immune response. IDA/IDL perception triggers both early and late immune responses: rapid calcium influx and production of reactive oxygen species (ROS) at the apoplast and induction of defense response genes, respectively (Butenko et al. 2014; Lalun et al. 2024). It is fascinating to speculate that the process of cell separation is inherently coupled with enhanced immunity, which can protect loose, exposed internal cells and tissues from pathogen invasion. Supporting this notion, *IDA* and *IDL6* are induced by pathogen elicitors, such as flg22 and chitin (Wang et al. 2017; Lalun et al. 2024). Furthermore, *IDL6* is rapidly induced by actual pathogen infection (*Pseudomonas syringae* DC3000), and its loss-of-function and overexpression confer susceptibility and resistance to Pst DC3000, respectively (Wang et al. 2017). These results highlight the roles of IDA/IDLs as phytocytokines to remodel the apoplastic environment to elicit an immune response (Hou et al. 2021). Notably, IDA can induce the expression of *PEP3*, which encodes a member of the phytocytokines PEPs with known roles in immunity and abiotic stress response (Nakaminami et al. 2018). Therefore, peptide hormones can coordinate multifaceted remodeling of developmental programs and amplify the response by cascades of phytocytokine gene expression.

#### CLAVATA3/Embryo surrounding region

A large family of *CLE* genes encodes small, secreted proteins with a conserved C-terminal sequence (known as the CLE domain), and at least 32 CLE members have been identified in Arabidopsis to date, with earlier studies appearing in *Plant Physiology* (Cock and McCormick 2001; Yamaguchi et al. 2016). The first *CLE* gene, maize *EMBRYO SURROUNDING REGION*, was identified through its specific expression in endosperm (Opsahl-Ferstad et al. 1997) (Fig. 1). Another founding member of the CLE family, Arabidopsis CLV3, and its cognate receptor CLV1 were originally identified from genetic mutations that confer the enlargement of shoot apical meristems (SAMs) and increased carpel numbers (Clark et al. 1997; Fletcher et al. 1999) (Fig. 1). The CLV3-CLV1 module and a transcription factor WUSCHEL form a negative feedback loop that maintains the homeostasis of stem cells (Brand et al. 2000). Given that CLV1 is an LRR-RLK (Clark et al. 1997), it was natural for the scientific community to predict that CLV3 functions as a ligand for CLV1. However, the path to identifying the mature form of the CLV3 peptide (MCLV3) and demonstrating its direct binding to the LRR domain of CLV1 was far from straightforward.

Initially, CLV3 was thought to function as a large, secreted protein, possibly as a multimer (Becraft 2002; Rojo et al. 2002; Lenhard and Laux 2003). However, as reported in *Plant Physiology*, the *CLV3* CLE domain alone was able to rescue the *clv3* phenotype when driven by the *CLV3* promoter, suggesting that bioactive mature CLE peptides may be processed (Ni and Clark 2006). Importantly, MCLV3 was successfully identified through mass spectrometry from Arabidopsis callus overexpressing CLV3 as a proline-hydroxylated dodecapeptide within the CLE domain (Kondo et al. 2006) (Figs. 1 and 2). Synthetic MCLV3 was able to confer the termination of the SAM, a phenotype identical to that of *CLV3* overexpressors, albeit at high concentration (Kondo et al. 2006). Identification of MCLV3 enabled researchers to demonstrate its direct binding to the CLV1 ectodomain by photoaffinity labeling of plasma membrane–localized, epitope-tagged CLV1 overexpressed in tobacco culture cells with radiolabeled MCLV3 peptide (Ogawa et al. 2008).

Posttranslational modification of CLE peptides is more complex than initially anticipated. Further mass spectrometry analysis determined a highly potent, mature CLV3 as a 13-amino-acid arabinosylated glycopeptide, where Hyp7 is modified with 3 L-arabinose ([Ara3]CLV3) (Ohyama et al. 2009) (Fig. 2). [Ara3]CLV3 exhibits high biological activity at a nanomolar level (Ohyama et al. 2009). Posttranslational modification of CLE peptides not only contributes to stable receptor binding but also protects the peptide from inadequate proteolytic processing. Andreas Schaller and colleagues reported in *Plant Physiology* that hydroxyprolination of the CLE peptide CLE40 prevents inappropriate proteolytic cleavage by subtilases that attenuates the bioactivity (Stuhrwohldt et al. 2020).

The studies of CLE peptides laid a ground to our understanding of how a large family of plant peptide hormones controls diverse aspects of development and physiology. Earlier, extensive, detailed expression and phenotypic analyses of individual *CLE* genes were performed in Arabidopsis and reported in *Plant Physiology* (Ni and Clark 2006; Jun et al. 2010). The CLE peptides are now classified into A-type, which can trigger SAM arrest and root growth inhibition, and B-type, which impacts vascular development (Yamaguchi et al. 2016). The B-type CLEs are represented by CLE41/42/44, collectively known as tracheary element differentiation inhibitory factor (TDIF), in which the mature peptide sequence was first determined from Zinnia suspension culture (Ito et al. 2006) (Fig. 1). The distinct biological activities of these CLE peptides are achieved through their corresponding receptors, while their intricate expression patterns likely contribute to functional redundancies and specificities. CLE41/42/44 (TDIF) are perceived by the tracheary element differentiation inhibitory factor receptor (TDR, also known as PHLOEM INTERCALATED WITH XYLEM [PXY]) (Fisher and Turner, 2007), whereas CLV3 is perceived by CLV1 as well as LRR-RLKs, BARELY ANY MERISTEM1 and 2 (BAM1/2), and RECEPTOR-LIKE PROTEIN KINASE2 (RPK2) (Deyoung and Clark 2008; Yamaguchi et al. 2016) (Figs. 1 and 3). CLV1 binds not only CLV3 but also several CLE peptides with different affinities, thus creating further complexities in signaling (Ogawa et al. 2008). Additionally, LRR-RLP CLAVATA2 (CLV2) and receptor-like cytoplasmic kinase CORYNE together likely transduce CLV3 signaling in parallel to CLV1 (Jeong et al. 1999; Muller et al. 2008) (Figs. 1 and 3). However, further biochemical studies failed to detect the direct binding of [Ara3]CLV3 to CLV2 and RPK2 (Shinohara and Matsubayashi 2015). More recently, 4 CLV3 INSENSITIVE RECEPTOR KINASES (CIKs), a family of LRR-RLKs closely related to SERKs/BAK1, were identified as coreceptors for CLV1 and RPK2 (Hu et al. 2018). Unlike SERKs/BAK1, however, the application of MCLV3 did not enhance the interaction of CIKs with CLV1 (Hu et al. 2018), suggesting that the receptor heterodimer formation is ligand-independent. Consequently, the molecular mechanisms governing CLE receptor complex assembly and signal activation remain an exciting area of investigation.

#### Epidermal patterning factor

EPFs and EPFLs are a family of secreted peptides that mediate a variety of developmental processes and tissue patterning in plants (Rychel et al. 2010) (Fig. 2). The founding member, EPF1, was identified as a regulator of stomatal spacing through a genome-wide overexpression study of CRPs in Arabidopsis (Hara et al. 2007). Its paralog EPF2 was also identified as an unannotated secreted protein that exhibits the highest coexpression coefficient with SPEECHLESS and TOO MANY MOUTHS (TMM), a master transcription factor of stomatal development and an LRR-RLP enforcing stomatal spacing, respectively (Nadeau and Sack 2002; MacAlister et al. 2007; Pillitteri et al. 2007; Hara et al. 2009) (Fig. 1). An independent, transcriptomic study also identified EPF2 (Hunt and Gray 2009). The discoveries of EPFs and EPFLs exemplify the power of functional genomics and coexpression network analysis to identify peptide hormones. The receptors for EPFs and EPFLs, ERECTA-family LRR-RLKs, are known to mediate developmental processes beyond stomatal patterning (eg stem elongation, shoot meristem and flower development, vascular patterning, and embryogenesis) (Shpak et al. 2004, 2005; Jiang et al. 2022; Bai and Torii 2026) (Fig. 1). However, genetic screens failed to identify *EPF/EPFL*s as upstream regulators of the *ERECTA* family outside of the context of stomatal development, likely owing to their complex redundancies. In fact, at least 2 *EPFLs* (*EPFL4* and *6*) and 4 *EPFLs* (*EPFL1*, *2*, *4*, and *6*) are required for inflorescence and shoot meristem architecture, respectively (Uchida et al. 2012; Kosentka et al. 2019).

Several new concepts have emerged from the studies of EPFs and EPFLs. First, unlike small peptides (eg systemin, PSK, CLEs, IDAs, and CIFs), EPFs and EPFLs are relatively large CRPs (∼50 amino acids of predicted mature peptides) with discrete structures reinforced by 2 or 3 intramolecular disulfide bonds (Kondo et al. 2010; Rychel et al. 2010; Ohki et al. 2011) (Fig. 2). Nevertheless, EPFs and EPFLs directly bind to the ERECTA-family and activate signaling (Lee et al. 2012), demonstrating that CRPs can also be perceived by LRR-RLKs. Thus, LRR-RLKs can perceive both small peptides and CRPs. This notion was further reinforced by the identification of the receptor for LURE, a CRP mediating pollen-tube guidance, which turned out to be an LRR-RLK, POLLEN RECEPTOR KINASE 6 (Takeuchi and Higashiyama 2016; Zhang et al. 2017).

Second, the EPF/EPFL family illustrates how endogenous ligands acting as receptor agonists and antagonists influence signaling and developmental outcomes (Ohki et al. 2011; Lee et al. 2015). EPF1 and EPF2 inhibit stomatal development, whereas EPFL9, also known as Stomagen, promotes stomatal development (Kondo et al. 2010; Sugano et al. 2010; Lee et al. 2012). Notably, EPFL9 peptide application confers excessive numbers of clustered stomata, a phenotype resembling that of the triple loss-of-function *erecta*-family mutants, hinting that EPFL9 may inactivate the ERECTA family receptors (Sugano et al. 2010; Lee et al. 2015). Indeed, biochemical and structural studies further showed that EPF1/2 bind to and activate the ERECTA-family receptors, whereas EPFL9 competitively binds to the same binding site and prevents the receptor activation (Lee et al. 2015; Lin et al. 2017) (Fig. 3). Because EPF1/2 and EPFL9 possess similar binding affinity to the ERECTA family, the local concentration of EPF1/2 and EPFL9 likely determines the final stomatal patterning (Lee et al. 2015; Lin et al. 2017). The exact mechanism of how EPFL9 prevents signal activation remains unclear. In any event, peptide hormone antagonists likely exist in other small peptide families and can be applied for structure-guided engineering for selective receptor inhibition (Song et al. 2013; Lee and De Smet 2016). This concept is further elaborated with the discovery of a systemin antagonist (Wang et al. 2025). Moreover, the interplay between peptide agonists and antagonists is important for RALF peptide-mediated immunity and pollen-stigma interaction (Stegmann et al. 2017; Lan et al. 2023). Therefore, peptide antagonism represents a broader regulatory principle in plant signaling.

Third, a notion of ligand discrimination by an accessory receptor has emerged from the studies of EPF/EPFL signaling. Specifically, LRR-RLP TMM discriminates different EPF/EPFL peptides to prevent signal bleed-throughs (Abrash et al. 2011). This system is especially powerful because the receptors for EPF/EPFLs regulate multiple developmental processes within the same organs (eg leaf, stem). Compared to stomatal lineage–specific EPF1/2, shoot/stem/vasculature regulators EPFL4/6 possess larger loop domains (Rychel et al. 2010). Biochemical and structural analyses confirmed that TMM reinforces the binding of EPF1/2 to the ERECTA family but dampens the binding of EPFL4/6 to ERECTA (Lin et al. 2017) (Fig. 3). Whether similar mechanisms exist for LRR-RLPs including CLV2, many of whose functions remain unclear, is an interesting future question.

## Discovery of plant peptide families: experimental approaches and technologies

As described in the previous discussion with selected case studies, complementary genetic and biochemical approaches have synergistically accelerated the discoveries of plant peptide hormone families with important functions. Earlier works identified secreted peptides through clear loss-of-function mutant phenotypes, including CLV3 (ie SAM enlargement and club-like siliques) and IDA (ie petals failing to detach after fertilization), or biochemical purification of bioactive compounds in the cases of systemin, PEP, and PSK. These 2 approaches sometimes converged on the identification of CIFs/SHENGEN2, peptide hormones for the surveillance of diffusion barrier integrity (Doblas et al. 2017; Nakayama et al. 2017).

Once key peptide ligand genes are identified, a search for their homologs is followed by comprehensive characterization of their expression patterns as well as loss-of-function and overexpression effects. This was the case for many peptide hormone families, including IDA/IDLs, CLEs, EPFs, and EPFLs. Notably, researchers have leveraged available genomic resources to mine previously unannotated small peptide genes. A *Plant Physiology* study developed the Arabidopsis unannotated secreted peptide database through an in silico screen (Lease and Walker 2006). Their pipelines filter open reading frames (ORFs) in 25 to 250 amino acid lengths, presence of a signal peptide, and absence of a transmembrane domain, which resulted in approximately 33,000 putative ORFs. A further survey for the presence of rice orthologs and additional filtering led to approximately 6000 ORFs, discovering the new members of the RALF peptide hormones (Lease and Walker 2006). A similar in silico approach by Ohyama et al. surveyed Arabidopsis ORFs of 70 to 110 amino acid lengths. Subsequent screening for the presence of the N-terminal signal peptide and exclusion of CRPs as well as known peptide family genes (eg PSK, PSY, RALF, and CLE), the researchers identified 110 putative novel peptide genes, including 5 members of the C-terminally encoded peptide (CEP) family (Ohyama et al. 2008). As the Arabidopsis genome annotation improves, researchers have reannotated peptide hormone families. For the case of CEP peptides, a later in-depth survey identified 10 additional Arabidopsis *CEP* peptide genes, making it a family of 15 (Delay et al. 2013; Roberts et al. 2013). Likewise, in-depth reannotation of RALFs was reported in *Plant Physiology* (Abarca et al. 2021).

Increasingly available genome information enabled not only the discovery of peptide hormones but also comprehensive matching of peptide-receptor pairs. For example, receptor libraries of RLKs have been produced using in planta–expressed RLK ectodomains fused with a covalent tag for rapid purification (Shinohara and Matsubayashi 2007). The receptor library successfully identified 3 LRR-RLKs as receptors of the RGF peptide hormones (Shinohara et al. 2016). Functional assays can also be combined for peptide-receptor matching. Recently, Yu et al. (2025) took advantage of the PEP receptor, which triggers ROS (Flury et al. 2013), and generated a library of chimeric RLKs with 140 LRR-RLK ectodomains from soybean fused with the PEP receptor cytoplasmic domain. By transiently expressing these chimeric receptors in planta and applying 55 synthetic peptides, the researchers identified 63 peptide-chimeric receptor pairs that can trigger ROS (Yu et al. 2025). Such a comprehensive pairwise matching screen can be scaled and creatively modified to expand the family of peptide hormones and their receptors to uncover their biological functions.

## Future perspectives

The past 3 decades have witnessed a remarkable expansion in the repertoire of plant peptide hormones and their cognate receptors, together with substantial progress in elucidating their biological functions across plant development, environmental responses, and immunity. As phylogenetic identifications and functional analyses of peptide hormone families expand to diverse plant species, it has become clear that peptide hormone families can be repurposed in a unique manner. For example, in *Plant Physiology*, a landmark paper reported that CLE peptides regulate legume nodulation (Mortier et al. 2010). Very recently, EPFL peptides have also been shown to promote nodule cell proliferation (Isidra-Arellano et al. 2026). Another great example is the repurposing of RGF peptides in the parasitic plant *Phtheirospermum japonicum* to drive organogenesis of haustoria (Fishman et al. 2025). As plant genome resources continue to expand, we will likely uncover a broader landscape of neofunctionalized peptide hormones.

The growing wealth of plant genome, crop pan-genome, and epigenome information, together with rapid advances in transgenic and genome-editing technologies for crop species, is enabling the targeted engineering of peptide hormones to improve crop productivity. For example, cis-regulatory design of *CLV3* allows precise control of tomato fruit size (Rodriguez-Leal et al. 2017). Genome editing of *EPF/EPFL* genes in cereal crops (rice, maize) has emerged as an effective strategy to enhance water-use efficiency and drought tolerance with minimal yield penalty (Caine et al. 2019). A recent *Plant Physiology* study extended this approach to maize through CRISPR-Cas9 editing of *EPFL9* paralogs in maize (Karavolias et al. 2023).

Another important future direction is to harness generative artificial intelligence (AI) to mine peptide hormone genes, pair with receptors based on the modeled structures, and further design artificial peptide hormones with higher specificity (eg for controlling only a specific subset of downstream signaling), a broad spectrum (eg for eliciting resistance to wide varieties of stresses/pathogens), or novel properties. AI protein design tools, such as RFdiffusion (Watson et al. 2023), could be utilized for de novo designs of peptide hormone binders to RLKs. Producing large amounts of peptides could be costly. Synthetic small molecules acting as peptide hormone mimics, with agonistic or antagonistic activities, could be an alternative option for easily scalable agricultural applications. Recently, small molecule screens identified a compound acting as a competitive antagonist of CLE peptide binding to its receptor (CLE9-BAM1) (Shinohara et al. 2019). Together with AI-assisted in silico platforms, such expansion of chemo-space for both peptide and nonpeptide peptide hormone mimics holds promise for understanding and tailoring plants with beneficial traits suited for the future climate.

Advances Box

- Since the discovery of systemin in 1991, our knowledge of plant peptide hormones has expanded tremendously, exemplifying how a scientific paradigm can shift so quickly.
- Biochemical purifications, genetic identifications based on phenotypes, and bioinformatic surveys leveraged by the available genome information synergistically advanced the knowledge of peptide hormone signaling.
- Peptide hormones are largely classified into small posttranslationally modified peptides and cysteine-rich peptides, both perceived by their cognate receptor kinases (while exceptions may exist).
- Peptide hormones play key roles in diverse processes in development, environmental response, and defense/immunity, and mediate tradeoffs for plant organismal fitness.

Outstanding Questions Box

- How can members of the same peptide hormone family function across diverse spatial ranges, from within the same cell and over short distances to long-distance root-to-shoot communication? What are the underlying mechanisms?
- How exactly do peptide processing and modification enzymes contribute to the activity, spatial range, and function of peptide hormones?
- What mechanisms ensure signal specificity while sharing the same sets of peptide hormones, receptors, coreceptors, and/or downstream components?
- Can we generate biosensors for visualizing the spatiotemporal dynamics and amplitude/frequency of peptide hormone perception and receptor activation?
- How can we leverage the power of artificial intelligence to help design peptide agonist/antagonist mimics and orthogonal peptide hormone-receptor systems to enhance plant resilience?

## Data Availability

All structural data supporting the findings of this review article are provided within the figure legends.
